# Adrenomedullin increases fibroblast-like synoviocyte adhesion to extracellular matrix proteins by upregulating integrin activation

**DOI:** 10.1186/ar3160

**Published:** 2010-10-14

**Authors:** Marie-Dominique Ah Kioon, Carine Asensio, Hang-Korng Ea, Benjamin Uzan, Martine Cohen-Solal, Frédéric Lioté

**Affiliations:** 1 INSERM UMR-S 606, Hôpital Lariboisière, Ambroise Paré Street, 75010 Paris, France; 2 Paris-Diderot University, Thomas Mann Street, 75013 Paris, France; 3 UFR Faculty of Medicine, Square Villemin, 75010 Paris, France

## Abstract

**Introduction:**

Rheumatoid arthritis (RA) is characterized by bone and cartilage invasion by fibroblast-like synoviocytes (FLSs). Adrenomedullin, a peptide with anabolic and antiapoptotic properties, is secreted by rheumatoid FLSs. Adrenomedullin also increases the expression of adhesion molecules in endothelial cells and keratinocytes. Here, we investigated whether adrenomedullin mediated FLS adhesion to extracellular matrix (ECM) proteins.

**Methods:**

FLSs were isolated from synovial tissues from RA and osteoarthritis (OA) patients. Plates were coated overnight with the ECM proteins vitronectin, fibronectin, and type I collagen (Coll.I). Adrenomedullin was used as a soluble FLS ligand before plating. We tested interactions with the adrenomedullin receptor antagonist (22-52)adrenomedullin and with the protein kinase A (PKA) inhibitor H-89, and inhibition of co-receptor RAMP-2 by siRNA. Cell adhesion was measured by using color densitometry. Activation of α_2 _and β_1 _integrins was evaluated by fluorescent microscopy; integrin inhibition, by RGD peptides; and the talin-integrin interaction, by immunoprecipitation (IP).

**Results:**

Adrenomedullin specifically increased RA-FLS adhesion to vitronectin, fibronectin, and Coll.I; no such effect was found for OA-FLS adhesion. Basal or adrenomedullin-stimulated RA-FLS adhesion was inhibited by (22-52)adrenomedullin, H-89, and RAMP-2 siRNA. Adrenomedullin-stimulated adhesion was inhibited by RGD peptides, and associated with α_2 _and β_1 _integrin activation. This activation was shown with IP to be related to an integrin-talin interaction and was significantly decreased by (22-52)adrenomedullin.

**Conclusions:**

Adrenomedullin-stimulated RA-FLS adhesion was specific for ECM proteins and mediated by α_2 _and β_1 _integrins. This effect of adrenomedullin was dependent on adrenomedullin receptors. These results support a new role for adrenomedullin in rheumatoid synovial fibroblast pathobiology.

## Introduction

Rheumatoid arthritis (RA) is a chronic inflammatory joint disease in which fibroblast-like synoviocytes (FLSs) invade the extracellular matrix (ECM) of cartilage and bone, causing destruction of both tissues. FLSs in patients with RA (RA-FLSs) differ from normal FLSs regarding both morphologic and biologic characteristics [1]. More specifically, RA-FLSs can adhere to ECM proteins [1,2] by a number of adhesion molecules.

Among the adhesion molecules expressed by FLSs, integrins interact with several ECM proteins, including collagen, fibronectin, and vitronectin. Integrins are heterodimeric proteins composed of two subunits, α and β. When integrins bind to their ligands, they undergo activation with conformational changes regulated by inside-out signals [3]. Changes in extracellular domain conformation are initiated by binding of talin to an integrin β cytoplasmic domain [4,5]. Binding of talin to integrin disrupts the salt bridge between the two chains, thereby activating the integrin molecule [6]. RA-FLSs overexpress the integrins α_1_β_1_, α_2_β_1_, α_3_β_1_, and α_v_β_3 _[7]. The activation and adhesion properties of RA-FLSs may be intrinsic to these cells or related to factors such as cytokines and other molecules produced by RA-FLSs. Adrenomedullin may be one such factor.

Adrenomedullin is a 52-amino acid peptide first identified in 1993 in human pheochromocytoma. This peptide contains two structures important for its activity: a loop of six amino acids formed by a disulfide bond between residues 16 and 21 and an amide bond on the C-terminal tyrosine residue [8]. The (22-52)adrenomedullin truncated peptide does not have the six-amino acid ring and can act as an antagonist of the adrenomedullin receptor, depending on the cell type and species. In FLSs, adrenomedullin binds to a heterodimeric plasma membrane receptor composed of the seven-transmembrane domain protein calcitonin receptor-like receptor (CLR) coupled to one of two receptor activity-modifying proteins (RAMP-2 or -3) [9]. CLR signal transduction is mediated through G protein-coupled adenylate cyclase (AC) and protein kinase A (PKA) pathways [10].

Several reports support a role for adrenomedullin in the pathogenesis of RA. Plasma adrenomedullin levels are higher in RA than in other inflammatory diseases (lupus and scleroderma), osteoarthritis (OA), and in normal individuals [11]. Furthermore, adrenomedullin levels in plasma and synovial fluid are higher in RA than in OA [12].

More recently, our group reported overexpression in RA-FLSs of adrenomedullin and its CLR/RAMP-2,3 receptor mRNA and protein, compared with FLSs from OA patients (OA-FLS) [10]. Adrenomedullin was secreted by RA-FLSs and may act as a soluble factor. Indeed, the adrenomedullin receptor was functional, because adrenomedullin stimulation led to intracellular cAMP production and to decreased FLS apoptosis triggered by various conditions through PKA. Moreover, (22-52)adrenomedullin competitively and dose-dependently blocked the antiapoptotic effect of adrenomedullin on RA-FLSs.

Interestingly, adrenomedullin has several other effects, depending on the cell type: it increases cell proliferation [13]; inhibits smooth muscle cell migration [14]; and stimulates human umbilical vein endothelial cell (HUVEC) proliferation and migration [15], as well as angiogenesis *in vitro *[15] and *in vivo *[16]. Adrenomedullin also induces the expression of adhesion molecules (intercellular adhesion molecule, or ICAM; and endothelial selectin, or E-selectin) at the surface of HUVECs [17] and human oral keratinocytes [18].

The objective of this study was to determine whether adrenomedullin was involved in the adhesion of RA-FLSs to ECM proteins of bone and cartilage.

## Materials and methods

### Synovial specimens

Synovial tissues were obtained under aseptic conditions from 11 RA and three OA patients undergoing total knee- or hip-replacement surgery or wrist synovectomy. All RA patients fulfilled the 1987 American College of Rheumatology criteria for RA [19], and all OA patients had joint pain with radiologic evidence of degenerative changes at surgery. Disease activity and medications at surgery were not recorded. All human sample-collection procedures complied with the Helsinki Declaration. According to French Law on human research (Law 2007-1110, article 1211-2), synovial sample collection (surgical waste) was authorized unless opposition from patients occurred; all patients were informed.

### Primary synovial fibroblast cultures

Synovial tissues were minced in HAM F-12 culture medium and then incubated overnight at 37°C with 1 mg/ml of type I collagenase (Sigma-Aldrich). After cell dissociation, the samples were filtered through a cell strainer. Cell suspensions and cultures were carried out as previously described [10]. Cell confluence and morphology were assessed throughout the experiments by phase-contrast microscopy, as described elsewhere [20]. All experiments were carried out by using primary synovial cells cultured between passages 3 and 6.

### Reagents

Human adrenomedullin (1-52) and (22-52)adrenomedullin were purchased from Bachem. PBS, trypsin-EDTA, and HAM F-12 were obtained from Invitrogen Life Technologies. Fetal calf serum (FCS) was from Dutscher; the same batch was used in all experiments. Fibronectin, vitronectin, Coll.I, bovine serum albumin (BSA), RAMP-2 siRNA, RGD and scrambled peptides, and the PKA inhibitor H-89 were purchased from Sigma-Aldrich. CD49b (α_2_) Fluorescein IsoThioCyanate (FITC)-conjugated antibody, CD29 (β1) phycoerythrin (PE)-conjugated antibody, PE Mouse IgG2a, and FITC Mouse IgG1 (isotype controls) were bought from BD Pharmingen. Paraformaldehyde (PFA) was purchased from Panreac.

### Adhesion assay

The wells of 96-well flat-bottomed plates were coated overnight at 4°C with 100 μl of three ECM proteins, at the following optimal doses defined in preliminary experiments: vitronectin, 0.1 μg/cm^2^; fibronectin, 0.01 μg/cm^2^; and Coll.I, 0.1 μg/cm^2^. BSA 0.1% and polylysin 0.1 μg/cm^2 ^were used as nonspecific controls. Wells were blocked with 0.1% BSA for 1 hour at room temperature (RT). Then FLSs previously cultured in HAM-F12 10% FCS were harvested with trypsin-EDTA, pelleted, and resuspended in HAM-F12 without FCS but with Ca^2+ ^and Mg^2+ ^to allow integrin activation.

To investigate the effect of adrenomedullin, we incubated FLS at RT for 1 hour with adrenomedullin at indicated concentrations. After washing, 10^5 ^cells were seeded in each well and incubated for different periods at 37°C. Each condition was done in quadruplicate. After incubation, unbound cells were removed along with the culture medium. Remaining adherent cells were colored for 15 minutes with 0.5% violet crystal and fixed with 20% methanol. Plates were washed twice with 300 μl of PBS 1X, and adherent cells were lysed with 1% SDS. Adhesion was quantified with spectrophotometric optic-density measurement at 550 nm (Dynatech MR5000).

To study the effects of the adrenomedullin receptor antagonist (22-52)AM, of the PKA inhibitor H-89, and of RGD peptide (integrin competitor), FLSs were preincubated with (22-52)AM or H-89 at 10^-8^, 10^-7^, or 10^-6 ^*M*, or with RGD (50, 100, 150 μg/ml; or scrambled peptide (150 μg/ml) as control) for 30 minutes and then treated with adrenomedullin or left untreated for 1 additional hour. The adhesion assay was performed as described earlier.

### Small interference RNA

To silence RAMP-2 expression in RA-FLS, an siRNA, ID number SASI_Hs01_00149404 [EBI:NM_005854], was used. Cells were transfected by using the INTERFERIN transfection reagent (Ozyme) according to the manufacturer's instructions. siRNA was diluted in transfection reagent and culture medium, and cells were incubated with siRNA (5, 10, and 20 n*M*) for 24 h before being processed for adhesion assay.

### Fluorescence microscopy

FLSs were incubated for 1 hour with 10^-7 ^*M *adrenomedullin and washed. Then, 10^5 ^cells were plated on eight-well LabTek uncoated coverslips (Nunc A/S) for 2 hours at 37°C. After incubation, nonadherent cells were removed along with the culture medium. The cells were fixed with 4% PFA in PBS. Nonspecific sites were blocked with 3% BSA in PBS for 1 hour at RT. For immunostaining, cells were incubated for 1 hour with both monoclonal AK-7 α2- FITC and HUTS-21 β1-PE antibodies (Mc Ab) diluted 1/40 in 3% BSA. As a negative control, cells were incubated with γ2-FITC and γ1-PE isotype control. The nuclei were then stained with DAPI for 5 minutes. Cells were mounted in fluorescent mounting medium (Dako) and viewed by using a fluorescence microscope (Nikon) interfaced with the software package Microvision Instrument.

Where indicated, FLSs were preincubated with 10^-6 ^*M *(22-52)AM for 30 minutes before being treated with adrenomedullin or left untreated for 1 hour and then processed for fluorescence microscopy, as described.

### Talin immunoprecipitation and Western blotting

At confluence, FLSs were harvested with trypsin-EDTA, pelleted, and resuspended in HAM-F12 without FCS. FLSs were incubated with adrenomedullin for 1 hour, and 10^5 ^cells were seeded in triplicate for 2 hours. After incubation, cell proteins were extracted in lysis buffer. After centrifugation, the protein content of the supernatants was determined by using the Pierce protein assay. For immunoprecipitation (IP), 1 μg of specific anti-talin antibody (Santa-Cruz) was incubated overnight at 4°C in a rotating device with Dynabeads Protein G (Dynal Biotech, Invitrogen) and 100-μg aliquots of protein lysates. The magnetic beads were placed on a magnet, washed 3 times, suspended in lysis buffer and 4× running buffer (40% glycerol, 8% SDS, 0.250 *M *Tris (pH 6.8), and 0.8 mg bromophenol blue), and denatured for 5 minutes at 95°C. The magnetic beads were placed on a magnet, and the denatured proteins were retained in 100 μl lysis buffer, whereas the beads were discarded. Aliquots (30 μl) were then subjected to electrophoresis resolved on 4-20% SDS-PAGE gel (Bio-Rad) and transferred onto Hybond polyvinylidene difluoride membrane (Amersham Biosciences). The membranes were incubated for 1 hour with 1× blocking buffer and then reacted overnight with anti-talin antibody diluted 1:500 in blocking solution. The membranes were washed and incubated with the appropriate peroxidase-coupled secondary antibody. The signal was visualized by using a chemiluminescence detection system (Bio-Rad).

### Statistical analysis

The data are reported as mean ± SEM for adhesion experiments (at least three separate experiments with different donors). Groups were compared by using ANOVA and *post hoc *tests (Fisher test). All values are normalized for the control value. *P *values less than 0.05 were considered significant.

## Results

### Adrenomedullin increases rheumatoid FLS adhesion to extracellular matrix proteins

**Figure 1:**
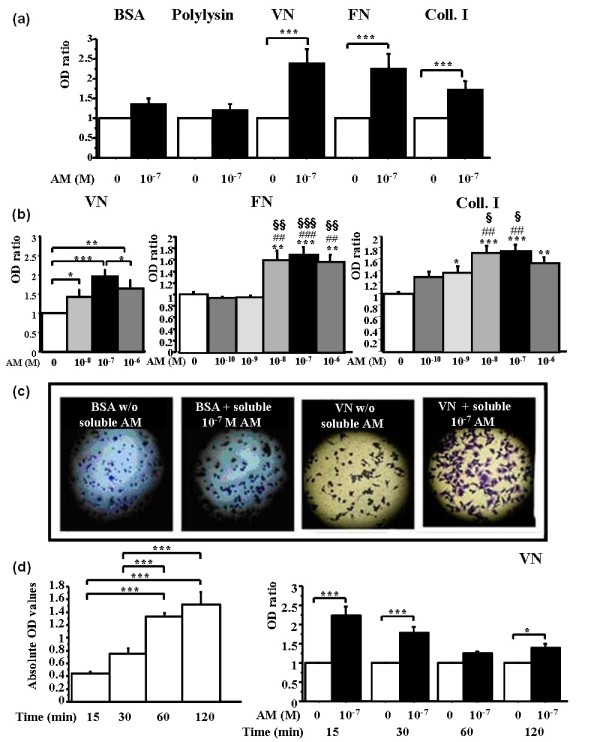
**Adrenomedullin significantly and dose-dependently increases the adhesion of RA-FLSs to vitronectin, fibronectin, and type I collagen**. RA-FLSs are resuspended in HAM-F12 without fetal calf serum and incubated for 1 hour with adrenomedullin (AM) at the indicated concentrations. Then 10^5 ^cells with or without adrenomedullin are seeded on 96-well plates previously coated overnight with the extracellular matrix proteins, bovine serum albumin (BSA), or polylysin. After 2-hour adhesion, nonadherent cells are removed, and the remaining RA-FLSs are stained with violet crystal for 15 minutes. The plates are washed, and the cells are lysed. Adherent cells are quantified by measuring absorbance at 550 nm with a spectrophotometer. **(a) **Effect of AM on RA-FLS adhesion to different surfaces. **(b) **The effect of AM is dose dependent. **(c) **Phase-contrast microscopy photographs demonstrating adhesion of RA-FLSs to BSA (negative control) and vitronectin with or without 10^-7 ^*M *AM. **(d) **Time course of RA-FLSs adhesion to vitronectin under basal conditions (left panel) and with AM (right panel). Bars indicate mean ± SEM. *P < 0.05; **P < 0.01; ***P < 0.0001 versus control; ^##^*P *< 0.01 and ^###^*P *< 0.0001 versus 10^-10 ^*M *AM; and, ^§§^*P *< 0.01 and ^§§§^*P *< 0.0001 versus 10^-9 ^*M *AM. Results are given as the ratio of optical density (OD) in each condition over control OD without AM and reported as mean ± SEM of three experiments (three different donors) performed in quadruplicate.

RA-FLSs were treated with 10^-7 ^*M *adrenomedullin for 1 hour and studied in a 2-hour adhesion assay, as described earlier. The 10^-7 ^*M *adrenomedullin significantly enhanced RA-FLS adhesion to all three ECM proteins (Figure 1a), with a 2.39-, 2.25-, and 1.65-fold increase for vitronectin, fibronectin, and Coll.I, respectively (all with *P *< 0.0001). No effect was seen with the negative controls (BSA and polylysin). These results suggest that adrenomedullin may specifically affect adhesion to FLS integrins, which are receptors of ECM proteins.

We evaluated RA-FLS adhesion after exposure to various adrenomedullin concentrations. The adhesion-enhancing effect increased in a dose-dependent manner: 1.41-fold with 10^-8 ^*M *(*P *= 0.04); twofold with 10^-7 ^*M *(*P *< 0.0001) of adrenomedullin; 1.6-fold at 10^-8 ^*M *(*P *= 0.0001); and 1.7-fold at 10^-7 ^*M *(*P *< 0.0001) on vitronectin and fibronectin, respectively (Figure 1b, middle panel). RA-FLS adhesion to Coll.I was dose-dependently increased by 1.4-, 1.7-, 1.8-, and 1.5-fold; *P *= 0.02, *P *< 0.0001, *P *< 0.0001, and *P *= 0.005 with 10^-9 ^*M *to 10^-6 ^*M *AM, respectively (Figure 1b, right panel). RA-FLSs exposed to adrenomedullin exhibited a spread-out shape on vitronectin after 2 hours, whereas the cells remained round on BSA (Figure 1c); no cell-to-cell adhesion was observed at higher-power field (not shown).

A time-course experiment was designed by using different incubation periods (15, 30, 60, and 120 minutes) with vitronectin only. Spontaneous RA-FLSs adhesion increased gradually with time from 0.43 OD at 15 minutes to 1.52 OD at 2 hours (Figure 1d, i). By contrast, the effect of adrenomedullin on RA-FLSs adhesion was already maximal after 15 minutes (2.2-fold increase; *P *< 0.0001). Nevertheless, this effect remained significant (1.4-fold increase, *P *= 0.02) after 2 hours (Figure 1d, ii). Furthermore, after 2 hours, adrenomedullin-stimulated RA-FLSs had a typical spread-out fibroblast-like shape, contrasting with the round, loosely bound shape seen after 15 minutes. In subsequent experiments, we used the 2-hour period associated with firmer attachment.

### The adrenomedullin effect is specific of rheumatoid FLSs adhesion

**Figure 2:**
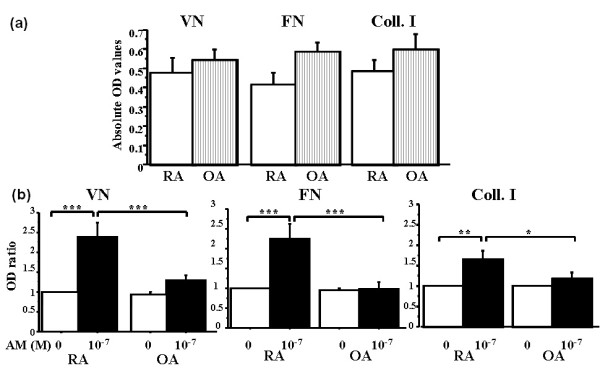
**Effect of adrenomedullin on adhesion of rheumatoid and osteoarthritic fibroblast-like synoviocytes (RA-FLSs and OA-FLSs, respectively)**. RA-FLSs or OA-FLSs are incubated for 1 hour with or without 10^-7 ^*M *adrenomedullin (AM) and seeded for 2 hours on 96-well plates coated with the ECM proteins. The number of adherent cells is assessed by using colorimetry. Bars indicate mean ± SEM. *P < 0.05; ***P < 0.0001. Results are given as the ratio of optical density (OD) in each condition over control OD without adrenomedullin and reported as mean ± SEM of three experiments (different donors) performed in quadruplicate. **(a) **Comparison of basal adhesion of RA-FLSs and OA-FLSs with vitronectin, fibronectin, and type I collagen. **(b) **Effect of 10^-7 ^*M *AM on RA-FLSs and OA-FLSs adhesion to vitronectin, fibronectin, and type I collagen.

Given previous evidence that RA-FLSs expressed more adrenomedullin and adrenomedullin receptors (CLR/RAMP-2 and CLR/RAMP-3) than did OA-FLS [10], we compared the effect of adrenomedullin on RA-FLSs and OA-FLSs. After 2 hours, basal adhesion to all three ECM proteins was not significantly different between RA-FLSs and OA-FLSs (Figure 2a). Adrenomedullin significantly increased RA-FLSs adhesion (*P *< 0.0001 for all comparisons) but had no effect on OA-FLSs adhesion (Figure 2b). This constitutes the first evidence that the adhesion-enhancing effect of adrenomedullin is specific of RA-FLSs, at least *in vitro*.

### The adhesion-enhancing effect of adrenomedullin is mediated by the adrenomedullin receptor CLR/RAMP

**Figure 3:**
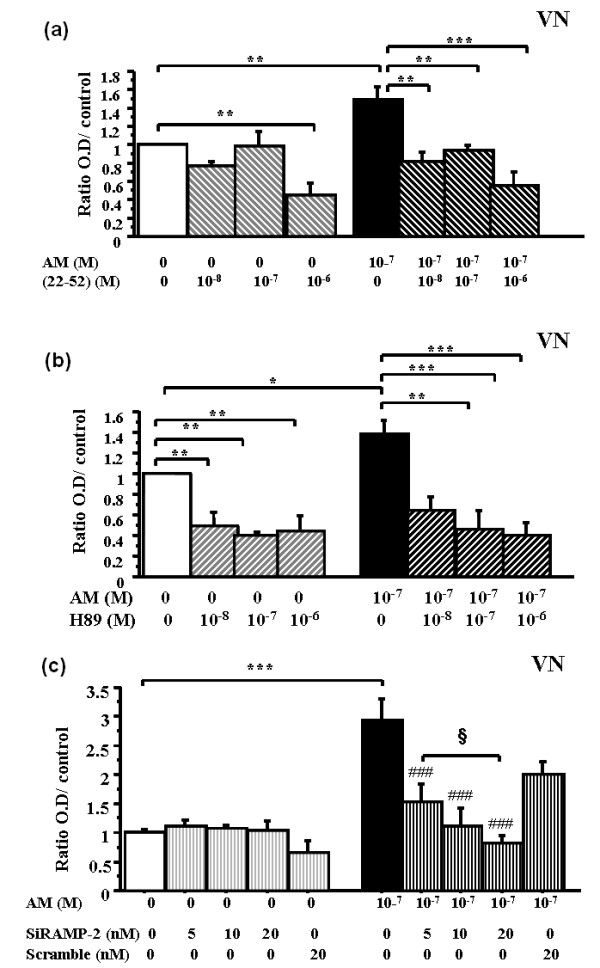
**Adrenomedullin exerts its action on RA fibroblast adhesion through CLR/RAMP receptors**. RA-FLSs were incubated for 30 minutes with various concentrations of (22-52)AM, or H-89, or for 24 hours with siRNA-RAMP-2 and then with or without soluble adrenomedullin (AM) for 1 hour. (22-52)AM, H-89, and siRNA RAMP-2 inhibited the adhesion-enhancing effect of AM. Bars indicate mean ± SEM. ***P < 0.0001; ^###^*P *< 0.0001 versus 10^-7 ^*M *AM, and ^§^*P *< 0.05. Results are given as the ratio of OD in each condition over control OD without AM and reported as mean ± SEM of two experiments (different donors) performed in quadruplicate. **(a) **Dose-dependent effect of (22-52)AM on basal and AM-stimulated RA-FLSs adhesion. **(b) **Dose-dependent effect of H-89 on basal and AM-stimulated RA-FLSs adhesion in each condition over OD of control. **(c) **Dose-dependent effect of siRNA RAMP-2 on AM-stimulated RA-FLSs adhesion in each condition over OD of control.

To investigate the role of adrenomedullin and its receptors in RA-FLSs adhesion, we preincubated the cells with (22-52)adrenomedullin, a specific antagonist of adrenomedullin receptors [10]. Without exogenous adrenomedullin, (22-52)adrenomedullin had no effect on RA-FLS adhesion as compared with control, even at (22-52)adrenomedullin highest concentration (10^-6 ^*M*) (decrease by 20%; *P *= 0.08) (Figure 3a). Adrenomedullin alone (10^-7 ^*M*) significantly increased RA-FLSs adhesion (1.4-fold; *P *= 0.009). This increase was significantly and dose-dependently inhibited by (22-52)adrenomedullin (decrease by 26%, 46%, 35%, 38%, and 71%; *P *= 0.02; *P *= 0.0002; *P *= 0.0004; *P *= 0.0002; and *P *< 0.0001 with 10^-10^, 10^-9^, 10^-8^, 10^-7^, and 10^-6 ^*M*, respectively) (Figure 3a).

To confirm further the role for the adrenomedullin receptor and downstream signaling molecules, we used the pharmacologic PKA inhibitor H-89. Basal RA-FLS adhesion was significantly and dose-dependently decreased by H-89 (0.51-fold to 0.66-fold; *P *= 0.005) (Figure 3b). Moreover, adrenomedullin-enhanced RA-FLSs adhesion (1.4-fold; *P *= 0.05) was significantly decreased by H-89 (0.36-fold to 0.6-fold; *P *= 0.002).

Finally, RAMP-2 siRNA enabled us to inhibit the AM co-receptor because it inhibited AM-induced adhesion by 48%, 62%, and 72% (*P *< 0.0001) with 5, 10, and 20 n*M *RAMP-2 siRNA, respectively (Figure 3c).

### Adrenomedullin-enhanced rheumatoid FLSs adhesion is linked to integrin activation

**Figure 4:**
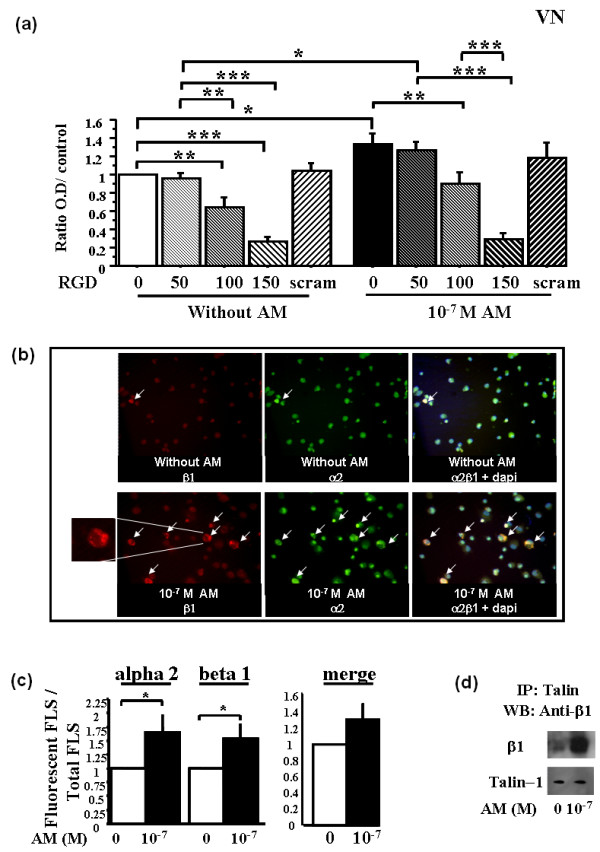
**Adrenomedullin significantly increases cell-surface expression of activated α**_**2 **_**and β**_**1 **_**integrins by rheumatoid fibroblast-like synoviocytes (RA-FLSs)**. RA-FLSs were incubated with 10^-7 ^*M *adrenomedullin (AM), or without AM as a control, for 1 hour. Bars indicate mean ± SEM. *P < 0.05; **P < 0.01; ***P < 0.0001. Inhibition adhesion study was done with RGD peptides and scrambled peptide for 30 minutes before AM stimulation. A fluorescence microscope was used for visualization of three sections and image capture. The mean ratio of fluorescent cells from these three sections was calculated as the ratio of the number of fluorescent cells over total cells counted by DAPI staining. Results are given as the ratio of fluorescent cells over control cells without AM and reported as the mean ± SEM of three experiments (three different donors) performed in triplicate. For IP, cell lysates from nonstimulated and AM-stimulated RA-FLSs were immunoprecipitated with anti-talin and then immunoreacted with anti-β_1 _with Western blot analysis (The figure is representative of two experiments done with two different RA-FLSs). **(a) **Dose-dependent inhibition of FLSs adhesion by using RGD peptides. Left panel, basal adhesion; right panel, AM stimulation. **(b) **Observation with fluorescence microscopy of the distribution of activated α_2 _and β_1 _integrin expression in RA-FLSs with or without 10^-7 ^*M *AM. Insert shows rim pattern or cytoplasmic membrane expression of activated integrin. **(c) **Effect of 10^-7 ^*M *adrenomedullin on the number of RA-FLSs expressing activated α_2 _and β_1 _integrins. **(d) **IP: Effect of 10^-7 ^*M *AM on the talin-β_1 _integrin interaction.

The specific and early effect of adrenomedullin on RA-FLS adhesion to ECM proteins suggested activation of latent adhesion molecules. Therefore, we evaluated the role of integrins in FLSs adhesion. RGD peptide (an integrin competitor) inhibited basal and AM-stimulated RA-FLSs adhesion in a dose-dependent manner, whereas the scrambled peptide had no effect (Figure 4a).

We then evaluated the effect of adrenomedullin on integrin activation. A number of monoclonal antibodies recognize "activation" epitopes (extracellular portions of the integrin molecule that are present only when its ligand is bound). We then used HUTS-21 and AK-7, which specifically recognize the activated β_1 _[21,22] and α_2 _integrin subunits, respectively. Adrenomedullin significantly increased the expression of both activated α_2 _(1.7-fold; *P *= 0.02) and β_1 _subunits (1.5-fold; *P *= 0.05) (Figure 4b, c). Co-localization of the activated α_2 _and β_1 _integrins was visible on the merged view (Figure 4b, right panel).

Moreover, we tested the hypothesis that adrenomedullin enhanced the interaction of talin with the β_1 _subunit, thereby causing integrin activation. The β_1 _subunit co-immunoprecipitated with talin, confirming that the two proteins are associated in a complex. Immunoblotting showed that adrenomedullin increased the talin-β_1 _interaction (Figure 4d).

### The receptor antagonist (22-52)adrenomedullin inhibits adrenomedullin-related integrin activation

**Figure 5:**
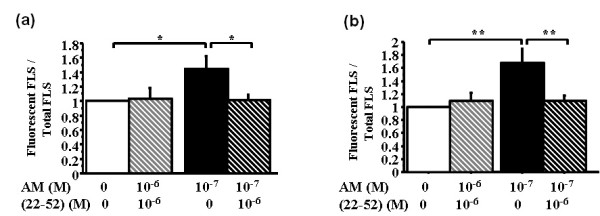
**(22-52)AM inhibits adrenomedullin-induced activation of α**_**2 **_**and β**_**1 **_**integrins on rheumatoid fibroblast-like synoviocytes (RA-FLSs)**. RA-FLSs were incubated for 30 minutes with various concentrations of (22-52)adrenomedullin (AM) or H-89 and then with or without soluble AM for 1 hour. Bars indicate mean ± SEM. *P < 0.05; **P < 0.01; ***P < 0.0001. A fluorescence microscope was used for visualization of three sections and image capture. The mean ratio of fluorescent cells from these three sections was calculated as the ratio of the number of fluorescent cells over total cells counted with DAPI staining. Results are given as the ratio of fluorescent cells over control cells without AM and reported as the mean ± SEM of three experiments (three different donors) performed in triplicate. **(a) **Mean fluorescence ratio of activated α_2_-subunit expression. **(b) **Mean fluorescence ratio of activated β_1_-subunit expression.

Because adrenomedullin exerted its effects on RA-FLS through the CLR-RAMP receptor, we pretreated RA-FLSs with 10^-6 ^*M *(22-52)adrenomedullin, for 30 minutes, before adding 10^-7 ^*M *adrenomedullin (Figure 5). After 2-hour adhesion, 10^-7 ^*M *adrenomedullin significantly increased the expression of the α_2 _and β_1 _subunits, 1.7-fold and 1.4-fold, respectively, compared with controls. The (22-52)adrenomedullin significantly decreased AM-stimulated α_2 _(Figure 5a) and β_1 _(Figure 5b) subunits expression to control level. However, (22-52)adrenomedullin alone had no effect on activated integrin expression, compared with control RA-FLSs.

## Discussion

**Figure 6:**
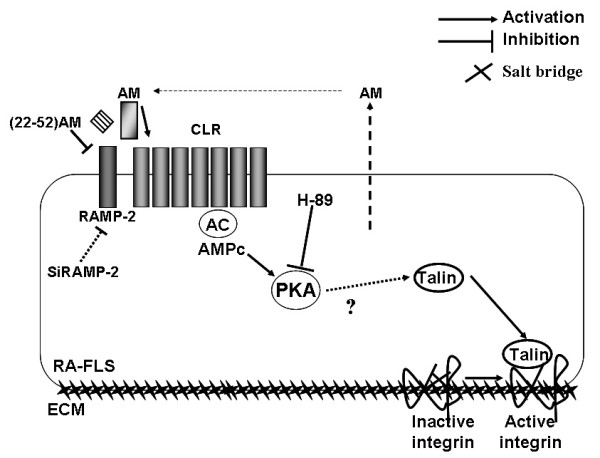
**Model of adrenomedullin-induced regulation of rheumatoid fibroblast-like synoviocyte (RA-FLS) adhesion by the CLR-RAMP/PKA pathway**. Adrenomedullin (AM) activates RA-FLS adhesion to the extracellular matrix proteins vitronectin, fibronectin, and type I collagen. The AM-receptor antagonist (22-52)AM and silencing of RAMP-2 inhibited AM-induced RA-FLS adhesion of CLR/RAMP results in activation of PKA, as assessed by its inhibition by H-89. AM triggers a talin-β_1 _chain interaction, disrupting the salt bridge between the α and β subunits and thereby activating the integrins, explaining the increase in RA-FLS adhesion.

This study provides the first evidence that adrenomedullin increases RA-FLS-integrin-dependent adhesion to several cartilage and bone ECM proteins by activating integrins, or at least their α_2 _and β_1 _subunits (Figure 6). Of note, this effect was observed only with RA-FLSs and not with OA-FLSs. Integrin activation was mediated by the adrenomedullin receptors CLR/RAMP, because it was inhibited by the receptor antagonist (22-52)AM, by inhibition of PKA, a downstream signaling molecule, and by inhibition of RAMP-2 by siRNA. Moreover, we obtained indirect evidence of integrin activation through a talin-dependent pathway.

Adrenomedullin increased RA-FLSs adhesion to the ECM proteins vitronectin, fibronectin, and Coll.I, which are abundant in the rheumatoid pannus. Increased levels of fibronectin have been found in rheumatoid synovial tissues [23-25]. Vitronectin was increased in the synovial lining layer from both OA and RA tissues, compared with normal tissues [26]. The expression of several integrin-receptor subunits is also increased *in situ*. Rheumatoid synovial tissues express high levels of the α_1 _to α_6 _subunits, and of the β_1_, β_3_, and β_4 _subunits, but low levels of the α_v _subunit [7,27,28]. The overexpressed integrins include α_5_β_1 _and α_v_β_3 _(the vitronectin receptor) and α_2_β_1 _(the Coll.I receptor). Coll.I accounts for 90% of the organic matrix of mineralized bone [29], and RA is characterized by bone invasion and erosion [30], a process preceded by attachment of RA-FLSs to the articular cartilage [31]. For these reasons, we selected fibronectin, vitronectin, and Coll.I for our study of RA-FLS adhesion.

Few studies have compared OA-FLSs and RA-FLSs adhesion to cartilage and bone ECM proteins [32]. Schedel *et al*. [32] showed that OA-FLSs and RA-FLSs adhered more strongly to types I, II, and VI collagens than to BSA, with no difference between OA and RA cells. These results are consistent with our finding that basal adhesion to Coll.I was not significantly different between RA-FLSs and OA-FLSs. Rinaldi *et al*. [7] showed that isolated RA-FLSs were significantly more adherent than normal FLSs to fibronectin, but also to other ECM proteins such as laminin and tenascin, a protein involved in RA synovium remodeling. However, no previous studies compared RA-FLSs and OA-FLSs regarding basal adhesion to vitronectin and fibronectin; we found no difference between RA and OA cells. We cannot assume a complete specificity of the adrenomedullin effect in RA because we have not tested other FLSs derived from psoriatic arthritis, as an example.

We found that adrenomedullin specifically increased RA-FLS adhesion to ECM proteins but had no effect on OA-FLS adhesion. This difference may be ascribable to the stronger expression of adrenomedullin receptor (CLR/RAMP2) mRNA and proteins by RA-FLSs than by OA-FLSs [10]. Moreover, our inhibition experiments using an adrenomedullin-receptor antagonist, an inhibitor of PKA, and one RAMP-2 siRNA confirmed the role for adrenomedullin receptors. It should be mentioned that no *in vitro *receptor binding kinetics compared (22-52)adrenomedullin with adrenomedullin.

Our findings suggest that the effect of adrenomedullin may be specific for adhesion molecules, such as integrins. The effect of adrenomedullin on RA-FLSs adhesion was already at its peak after 15 minutes, when adhesion was loose, whereas after 2 hours, the RA-FLSs exhibited the typical spreadout fibroblast-like appearance. In our attempt to demonstrate early adhesion-molecule expression, we selected integrins, because these are presynthesized in an inactive form and expressed at the membrane cell surface, and they are natural receptors for ECM proteins. With the specific antibodies AK7 and HUTS-21, we found that adrenomedullin induced integrin activation through a mechanism involving the adrenomedullin receptors. Integrin-mediated cell adhesion has been shown to depend on cAMP/PKA [33].

We studied only the α_2 _and β_1 _integrin subunits, which are involved in cell adhesion, not only to Coll.I and fibronectin, but also to other ECM molecules of interest, such as type II collagen [7]. The signaling pathways leading from CLR-AC-PKA activation to integrin activation are unknown. However, we hypothesized that talin, a major integrin activator, was activated by adrenomedullin. As siRNA would inhibit not only adrenomedullin-stimulated adhesion, but also basal adhesion, we chose talin IP followed by integrin β_1 _immunoblotting to investigate the interaction of the two proteins with adrenomedullin. We found that adrenomedullin stimulated integrin activation by increasing this talin-β_1 _interaction. Moreover, by using the adrenomedullin-receptor antagonist (22-52)AM, we were able to confirm the role for adrenomedullin in α_2 _and β_1 _integrin activation.

Previous studies have established that adrenomedullin activates adhesion molecules in other cell types. With HUVECs, adrenomedullin induces cell-surface expression of E-selectin, vascular cell adhesion molecule (VCAM-1), and ICAM-1 [17], through adenylate cyclase activation. These studies investigated only the effect of adrenomedullin on adhesion-molecule expression, whereas we used a reproducible functional adhesion assay to study FLSs adhesion in addition to integrin activation.

Adrenomedullin also increased capillary cell activation and neoangiogenesis in various animal models of tissue repair or tumor growth [34,35]. Angiogenesis occurs in the rheumatoid pannus and is an early event in collagen-induced arthritis [36]. Adrenomedullin [37] and (22-52)adrenomedullin [38] can stimulate or inhibit the spread of metastases *in vivo*, respectively. Cell adhesion is the first event in a multistep process and is followed by migration and invasion in angiogenesis, tumor growth, and invasiveness. We have obtained preliminary evidence supporting a role for adrenomedullin in increasing RA-FLSs migration in a two-dimensional system (Asensio and Ah Kioon, unpublished data). Overall, adrenomedullin may contribute to cell adhesion, migration, proliferation, and reduced apoptosis. These events are involved not only in pannus progression but also in tumor development, indicating a new role for adrenomedullin.

## Conclusions

In this work, we showed that soluble adrenomedullin increases RA-FLSs adhesion to various ECM proteins expressed in synovial tissue, cartilage, or bone. This effect is mediated by the adrenomedullin receptor CLR, and it does not occur with OA-FLSs, which express fewer adrenomedullin receptors [10]. This adrenomedullin-dependent RA-FLSs adhesion is ascribable to activation of the talin-β_1 _interaction followed by integrin activation (Figure 6). Thus, adrenomedullin may participate in the regulation of RA-FLSs and therefore to some extent in the pathogenesis of RA.

## Abbreviations

AC: adenylate cyclase; BSA: bovine serum albumin; CLR: calcitonin receptor-like receptor; Coll.I: type I collagen; ECM: extracellular matrix; FCS: fetal calf serum; FITC: fluorescein isothiocyanate; FLS: fibroblast-like synoviocyte; HUVEC: human umbilical vein endothelial cell; ICAM: intercellular adhesion molecule; IP: immunoprecipitation; OA: osteoarthritis; PE: phycoerythrin; PFA: paraformaldehyde; PKA: protein kinase A; RA: rheumatoid arthritis; RAMP: receptor activity-modifying proteins; RT: room temperature; VCAM-1: vascular cell adhesion molecule.

## Competing interests

The authors declare that they have no competing interests.

## Authors' contributions

MDAK was responsible for the conception and design, acquisition of data, analysis and interpretation of data, and drafting of the manuscript; CA, for acquisition of data and drafting of the manuscript; HKE, for conception and design, analysis and interpretation of data, and drafting of the manuscript; BU, for acquisition of data, analysis and interpretation of data, and drafting of the manuscript; MCS, for analysis and interpretation of data and drafting of the manuscript; FL, for conception and design, analysis and interpretation of data, drafting of the manuscript, and supervision. All authors read and approved the final manuscript.
